# Strengths and Weaknesses of Physiotherapy in the Daily Work of an Intensive Care Unit: A Qualitative Study

**DOI:** 10.3390/jcm14072283

**Published:** 2025-03-27

**Authors:** Anabel Solares-Mogollón, Rubén Cuesta-Barriuso

**Affiliations:** 1 Faculty of Medicine and Health Sciences, University of Oviedo, 33006 Oviedo, Spain; anabelsm64@gmail.com; 2 Department of Surgery and Medical-Surgical Specialties, University of Oviedo, 33006 Oviedo, Spain; 3 InHeFis Research Group, Instituto Asturiano de Investigación Sanitaria (ISPA), 33011 Oviedo, Spain

**Keywords:** intensive care unit, physiotherapy, strengths, barriers, qualitative study

## Abstract

**Objectives**: To describe the strengths and barriers of administering a physiotherapy treatment to patients admitted to an intensive care unit. **Methods**: Qualitative interpretative description study. Twenty-one health professionals working in an intensive care unit in two referral hospitals were recruited in the study. Each personal interview began with open-ended questions and then continued with more interview-inspired questions. All healthcare professionals gave their views on their knowledge, perceptions and observations of the strengths and weaknesses of physiotherapy in the treatment of patients admitted to this unit. **Results**: The analysis highlighted four main topics: (i) knowledge of the role of physiotherapists at the ICU; (ii) benefits of physiotherapy for patients and in a multidisciplinary team environment; (iii) challenges and proposals for improvement in interprofessional collaboration; iv) needs for the implementation of physiotherapy. **Conclusions**: This study analyzes the opinion of intensive care unit professionals regarding the strengths and barriers of physiotherapy in these units. Healthcare professionals highlight the importance of early physiotherapy treatment, the insufficient number of physiotherapists in these units and the benefits of physiotherapy in the respiratory and functional improvement of patients. The main perceived barriers are communication between professionals and the need to reduce the ratio of patients per physiotherapist.

## 1. Introduction

Intensive care units (ICUs) are the areas of the hospital environment responsible for providing health care to critically ill patients suffering from disorders of such magnitude that their lives are at risk [1]. The healthcare role played by intensive care units generates a high consumption of resources, being a stressful and emotionally charged area of work for healthcare professionals [2]. ICU distribution depends on the size of the hospital, the professionals working there and the technology available, with the optimal number of beds per unit being from 8 to 12 [3]. The multidisciplinary team that works in these units is made up of intensivist doctors, nurses, physiotherapists, nursing assistants and wardens. There should be good communication, coordination and trust between them to work together towards a common goal, the recovery of the patient [1,2,3].

Patients may reach the intensive care unit due to an acute illness, an invasive intervention or a polytrauma that puts one or more vital functions at risk [3]. These patients need continuous assistance and specialized care 24 h a day in a specific area [1]. There are varied pathologies, including those affecting the cardiovascular, respiratory, renal, metabolic, hepatic or cerebral function [3]. Patients admitted to the ICU have high chances of developing physical, functional, cognitive and mental problems, resulting in a deterioration of the patient’s quality of life [1,4].

Many patients who survive their admission to intensive care units have long-term adverse effects, associated with immobilization and prolonged inactivity during their stay, with a decrease in their physical function that affects their quality of life [5,6]. Critical care has evolved over recent decades. It has moved from a model of deep sedation and rest to a model of awakening and early mobility. Physiotherapists are taking on more prominent roles in the clinical and research approach in this area through increasingly broad interdisciplinary collaboration [7]. Physiotherapy is part of the multidisciplinary team that is responsible for the treatment of these patients [8]. Early rehabilitation of patients in intensive care units has shown beneficial effects on prognosis and quality of life, improving the degree of functionality and the development of activities of daily living at discharge [9,10]. Physiotherapy includes several aspects: pulmonary rehabilitation, early mobilization, cardiovascular exercise, muscle strengthening and passive or active mobilizations, all of them focused on achieving the same goal, the physical and functional recovery of patients [9,11].

Because of the increasing presence of physiotherapists in intensive care units, several studies have established agreed-upon minimum standards of clinical practice for physiotherapists working in intensive care units [12,13]. However, the use of specific resources can vary considerably depending on the type of intensive care unit and the type of hospital, with the working conditions of physiotherapists varying considerably in terms of workload, training and experience [14]. A recent study has shown how patients perceive the personalization of physiotherapy in the ICU, despite the lack of collaboration during the initial phase of their stay in the ICU [15].

Intensive care has evolved a lot in recent years with the responsibility of improving patient-centered care [16]. In this way, the important role played by physiotherapists has gained value, so their presence in intensive care units should be ensured in order to achieve early, comprehensive and efficient rehabilitation [11]. Currently in Spain, in pediatric ICUs, the physiotherapist-per-bed ratio is 1:8, while in adult ICUs, it is 1:13 [17]. However, the European Society of Intensive Care Medicine (ESICM) recommends a ratio of 1:5, seven days a week [3].

Although the presence of physiotherapists is not new, multidisciplinary work requires coordination and communication among the healthcare professionals working in the ICU. Therefore, knowing the barriers and limitations of this joint work, as well as its strengths, from the perspective of healthcare professionals with proven experience can help estimate which measures to implement even when teamwork has already been going on for years. Therefore, the main aim of this study was to describe the strengths and barriers of administering physiotherapy treatment to patients admitted to an intensive care unit as perceived by healthcare professionals with experience in these units.

## 2. Materials and Methods

### 2.1. Study Design

The study design is a qualitative observational study using personal interviews. Based on the “right to health” model [18], this qualitative study has been designed to explore the strengths and barriers perceived by ICU professionals regarding the physiotherapy approach in this unit.

### 2.2. Research Paradigm

The Hopscotch Model [19] was used to minimize the drawbacks of the paradigmatic approach to teaching research methodology, maximizing the connection between theory and practice. This design is based on nine proposed steps: (i) description of the paradigmatic position; (ii) definition of the objectives; (iii) description of the conceptual framework; (iv) definition of the research design; (v) definition of the research questions; (vi) proposal of data collection techniques; (vii) proposal of analysis strategies; (viii) definition of the reliability and veracity strategies; and (ix) description of the ethical principles. Once these steps are completed, the tool creates the research design [20].

### 2.3. Participants

The subjects included in the study had to answer the questions posed by the principal investigator with open or closed answers through a questionnaire created for this purpose. The inclusion criteria were as follows: (i) professionals from an intensive care unit, (ii) with at least 2 years clinical experience and (iii) who currently work or have worked in coordination with physiotherapists within the intensive care unit. The exclusion criteria for participation were (i) professionals not familiar with the usual physiotherapy therapy in an intensive care unit and (ii) medical interns in their first year of training.

### 2.4. Ethical Considerations

The confidentiality and anonymity of all the data obtained were ensured, complying with the ethical aspects contained in the Declaration of Helsinki. The research project was approved by the Research Ethics Committee of the Principality of Asturias (CEImPA code 2024.026). The study was registered in the international database of clinical records www.clinicaltrials.gov (https://clinicaltrials.gov/study/NCT06407076; 9 May 2024).

### 2.5. Sample Size

Although an exact methodology has not been established to calculate the number of participants required for data saturation in a qualitative research, different studies have reported how to achieve data saturation after conducting interviews with a sample size of 6 [21], 12 [22] or 17 [23] participants. Seven subjects were interviewed in each group of health professionals in order to achieve data saturation with the information provided by those interviewees, obtaining sufficient information on the topics for thematic analysis.

### 2.6. Method and Data Collection

All data were collected through interviews with the participants. Personal interviews were conducted between May 2024 and February 2025, in a room of the Intensive Care Unit of the Central University Hospital of Asturias and University Hospital of Burgos. The data were analyzed using the thematic analysis approach according to the method proposed by Braun and Clarke [24].

All the interviews were conducted by the same researcher. The interviewer gathered general demographic information before proceeding with the interviews. Each personal interview started with open-ended questions and then continued with more interview-inspired questions. Key concepts related to physiotherapy, its main strengths and weaknesses and the approach thereto as a health sciences discipline in an intensive care unit were collected. The interview guide (Table 1) included questions about healthcare professionals’ experiences of the work of physiotherapists in the intensive care unit, their experiences of collaboration with physiotherapists and barriers and strengths to successful multidisciplinary working. On average, each personal interview lasted approximately 30 min.

### 2.7. Data Analysis

The quantitative data collected on the characteristics of the patients were captured and analyzed in SPSS (Version 26) for Windows (IBM Company, Armonk, NY, USA) using descriptive statistics (median). The analyses of the qualitative variables were presented as frequencies.

All the interviews were read and analyzed word by word, verifying and correcting the resulting transcripts. Independently, the initial ideas for coding were recorded. The data were coded inductively, allowing data codes to emerge. The interpretative phenomenological analysis was completed by a different researcher from the one who conducted the surveys, after the transcripts were reviewed and collated for accuracy. The data were analyzed inductively by this researcher according to the principles of interpretative content analysis, using a systematic process to summarize and categorize the data and then generate subcategories, categories and topics.

### 2.8. Soundness of the Results

Regarding the rigor of the results, the interpretive description considers that the credibility of the results derives mainly from how specific analytical decisions are presented and contextualized [25]. However, traditional concepts of reliability may contribute to the credibility of this study [26]. This study conforms to traditional conventions of interpretive description, citing and being faithful to key publications with a focus on actionable results (credibility), including respondents’ interpretations and reflections (reliability), being transparent about analytic decisions (confirmability), recruiting a sample for the purpose of ensuring different levels of clinical expertise within an intensive care unit (authenticity) and providing a complete picture of the sample (transferability) [26].

## 3. Results

A total of 21 health professionals participated in the interviews. Most of the participants were women (71.43%), with an average age of 38.33 (±5.58) years and work experience in an intensive care unit of 9.80 (±4.61) years. Only 28.57% had previously worked in an ICU without the participation of physiotherapists in the management of admitted patients. Table 2 shows the main descriptive characteristics of the health professionals who participated in the study depending on the professional category.

Based on the analysis of the interviews, four main topics were generated: (i) knowledge about the role of physiotherapists in the ICU; (ii) benefits of physiotherapy for patients admitted to the ICU and in the multidisciplinary team environment; (iii) challenges and proposals for improvement in interprofessional collaboration; and (iv) needs for the implementation of physiotherapy in the ICU. Moreover, there is a fifth section of free-response remarks and comments.

Although each topic is presented reflecting previous professional experiences, recommendations and expectations, they are interconnected. The topics are discussed below in terms of how they connect to each other, along with the sub-topics.

### 3.1. Knowledge About the Role of Physiotherapists in ICU

Healthcare professionals expressed different views on the roles of physiotherapists in the treatment of patients admitted to ICU. These views are structured in two sub-topics: (1) when should the physiotherapist’s work with the ICU patient begin and (2) which is the physiotherapist’s role with these patients.

#### 3.1.1. When Should the Physiotherapist’s Work with the ICU Patient Begin?

Most of the participants stated that the physiotherapist’s work should start early at the time of the patient’s admission to the ICU as long as the latter is stabilized. In addition to this widely supported statement, health professionals also noted the need for physiotherapists after extubation and in view of the potential development of polyneuropathies in these patients.

#### 3.1.2. Which Is the Physiotherapist’s Role with These Patients?

Practically all health professionals were in agreement about the knowledge of the physiotherapist’s functions regarding the development of respiratory techniques and motor work in patients. Reference was also made, although to a lesser extent, to the work of physiotherapists in the mobilization of bedridden patients and the prevention of polyneuropathies.

### 3.2. Benefits of Physiotherapy for Patients Admitted to the ICU and in the Multidisciplinary Team Environment

In the analysis of the benefits provided by physiotherapy in an ICU, two sub-topics are described: (1) physiotherapy-based benefits in the therapeutic approach of the ICU patient and (2) benefits that the professional considers most important to promote multidisciplinary work in these units.

#### 3.2.1. Physiotherapy-Based Benefits in the Therapeutic Approach of the ICU Patient

The shorter the length of stay in the ICU, early mobilization and being able to implement extubations earlier were the clinical issues most highlighted by the majority of the healthcare providers interviewed. Likewise, almost half of the professionals emphasized the improvement of the patients’ independence and their self-esteem as a result of the clinical improvements that physiotherapy provides.

#### 3.2.2. Benefits That the Professional Considers Most Important to Promote Multidisciplinary Work in These Units

One aspect highlighted by professionals to facilitate multidisciplinary work is the need for more communication between themselves and more physiotherapists in the ICU, emphasizing the need for multidisciplinary clinical sessions. Likewise, they indicate that the main facilitating factor of multidisciplinary work would be the presence of more physiotherapists and a wider schedule, availing greater coverage in morning and afternoon shifts.

### 3.3. Challenges and Proposals for Improvement in Interprofessional Collaboration

All multidisciplinary work presents a series of challenges among health professionals. In this topic, the results have been analyzed through two sub-topics: (1) challenges experienced between ICU professionals and physiotherapists and (2) proposals to improve multidisciplinary collaboration in the ICU.

#### 3.3.1. Challenges Experienced Between ICU Professionals and Physiotherapists

Most of the interviewees do not indicate any previous experience identified as a challenge in collaborating with physiotherapists. Communication problems with the nursing team have sometimes been observed as an obstacle to better collaboration between the two health specialties. In addition, the need to get in touch directly and urgently is an aspect highlighted by medical professionals, as well as the possibility of autonomous management of respirators by physiotherapists.

#### 3.3.2. Proposals to Improve Multidisciplinary Collaboration in the ICU

Proposals include the improvement of communication between professionals, including the performance of joint clinical sessions. One aspect, pointed out by many of the interviewed professionals, is the need for more hours of physiotherapy intervention in the intensive care unit.

### 3.4. Needs for the Implementation of Physiotherapy in the ICU

When health professionals were consulted on what issues or aspects would be necessary to improve the implementation of physiotherapy in the ICU, an increase in human resources was one of the most demanded. The need for more staff, with more hours of dedication, was mostly indicated. A lower ratio of patients per physiotherapist and the provision of better and more adapted material were additional aspects pointed out by the interviewees.

### 3.5. Free-Response Remarks and Comments

At the end of the interview, the health professionals were able to present some final comments, and the assessment of the medical and nursing professionals was very positive regarding the work of physiotherapists and their importance in the approach to these patients.

The topics and sub-topics, as well as support appointments and healthcare professional coding, are shown in Table 3.

## 4. Discussion

This study aimed to determine the strengths and barriers of the administration of physiotherapy treatment in patients admitted to an intensive care unit. The observed results reinforce the importance of physiotherapy in the daily work in an intensive care unit according to the health professionals who work there. All the healthcare providers surveyed consider the presence of physiotherapists in these units necessary, and most of them consider the current number of physiotherapists to be insufficient.

Health professionals consider it important to start physiotherapy early as part of the treatment of critically ill patients. This is consistent with scientific evidence [27] that has shown how early progressive mobilization should be implemented as a priority in all adult intensive care units, as it is an area of clinical concern for ICU physiotherapists.

The administration of physiotherapy treatment within 72 h after admission can improve physical and cognitive function and functional prognosis, helping in the prevention of polyneuropathies. These improvements are in addition to the improvement of mobility, promoting the development of activities of daily living and a better perception of quality of life of these patients [28]. The functions of physiotherapists in intensive care units are widely recognized by all the professionals of the multidisciplinary ICU team, highlighting the administration of motor and respiratory physiotherapy techniques, for which evidence has been supported [9].

Among the main benefits observed by health professionals, the decrease in the length of stay in the intensive care unit and greater success in extubations are highlighted. After the first 6 h of connection to mechanical ventilation, there is a decrease in diaphragmatic strength, with the situation becoming more complicated in proportion to the connection time. Thus, patients who require this support suffer from weakness of the respiratory muscles, and this is one of the main clinical factors of failure of the weaning process from mechanical ventilation until patients are able to breathe on their own [29].

The medical and nursing professionals emphasize, almost unanimously, the physiotherapist’s role regarding the development of respiratory physiotherapy and motor work techniques in patients admitted to the ICU. Early mobilization together with inspiratory muscle training can reduce hospital stay and mortality during admission, preserving muscle strength, reducing the time of mechanical ventilation and helping successful weaning [29,30].

Multidisciplinary work is perceived as a reality among the study participants, although communication barriers may arise between professionals. In order to eliminate these barriers, improving the care provided in these units, the health professionals propose to improve communication by conducting joint clinical sessions that promote better coordination. This multidisciplinary planning of the steps to be followed with patients admitted to the ICU can facilitate the implementation of common and realistic objectives for the best patient approach [31]. The results of this study are consistent with those described by Tadyanemhandu et al. [32] regarding the importance of multidisciplinary teamwork and communication of professionals working in the ICU. Similarly, clarification of roles among multidisciplinary team members, development of expertise and skills training are essential. Similarly, potential areas of conflict over competencies between different healthcare professionals must be considered. These situations of potential conflict require close and effective collaboration and communication within the team. This can be observed, for example, between the nursing and physiotherapy teams. The two health care specialties may have different approaches to the prevention of pressure ulcer development.

One way to improve interprofessional collaboration emphasized by many of those interviewed is by increasing the presence of physiotherapists in intensive care units. In addition to the healthcare improvements for patients, an increase in physiotherapy staff and a greater hourly dedication of physiotherapists with the establishment of more morning and afternoon shifts would promote better integration of said professionals in these units. Training physiotherapists in the management of ventilators and the implementation of localization systems in the face of the need for urgent actions (for example, by means of a pager) are two aspects that, according to the interviewed professionals, would help to complement the two-way information exchange between health professionals. The promotion of physiotherapy should include the timely identification of suitable candidates with established safety standards, the coordination of evidence-based interventions with specific sedation regimens and the periodic evaluation of mobilizations and functional outcomes at the time of discharge from the ICU [33].

The main barriers described by healthcare providers are the limited number of physiotherapists in the unit and the limited treatment time they can devote to patients. These staffing constraints have already been described in previous studies [31]. The implementation of physiotherapy would be reinforced by increasing the staff and the available material and establishing physiotherapy permanently in the ICU. Several studies support the relationship between the number of physiotherapists working full time in the ICU and the timely performance of early mobilization techniques when patients are awake and medically stable [34,35]. Although the efficacy of interventions aimed at improving physical functioning after an ICU stay through in-hospital physiotherapy [36] or the implementation of progressive mobilization programs by participating in physical activities [36] has been described, the limited implementation of physiotherapists in ICUs is a considerable limitation. Therefore, the feasibility of creating full-time positions for ICU physiotherapists in hospitals needs to be further explored [32].

The implementation of a true multi-professional treatment of patients, together with the support of their families, in the ICU requires a proportionate number of health professionals [37]. Another aspect to help remove these barriers is improving the physiotherapist-per-bed ratio and increasing the treatment time for each patient. This aspect highlights the importance of implementing the 1:5 ratio recommended by the European Society of Intensive Care Medicine [3], which is above the healthcare reality of hospital centers in our country, where it is considerably lower.

The work and performance of physiotherapy professionals are highly valued by all other health professionals in the intensive care unit, recognizing the work they perform and its importance in the evolution of patients. Our results regarding the relevance of the role of the physiotherapist coincide with those described in a recent study [38] where it is pointed out that patients leaving the ICU are at risk of remaining immobilized because ward nurses consider mobilization too complex to be carried out without the support of physiotherapists. Close collaboration between physiotherapists and nurses leads to feelings of security, increased autonomy and alertness among patients, improving their recovery and leading to earlier discharge from hospitals [39].

### Study Limitations

Although the recruitment of professionals from each specialty was established, the number of physiotherapists who usually work in the intensive care unit and who met the selection criteria was lower. In the same way, the development of a more comprehensive semi-structured interview with additional questions not included regarding other aspects or concerns would make the results more substantial. Multi-professional team collaboration during mobilization has been shown to improve patient satisfaction [33]. The opinions and experiences of patients and their relatives should be included, monitored and given feedback in clinical practice in order to continuously improve ICU care [40]. Another limitation that needs to be considered is the evaluation of experiences of health professionals from two hospitals in the same country where access to healthcare is universal. Therefore, these results should be contrasted with those of other studies where the national health system, medical coverage and access to the health system are different. One source of bias in the present study is the inclusion criterion whereby only healthcare professionals working with physiotherapists were included in the study. The views of those professionals who do not work with physiotherapists in an ICU could provide essential input to understanding the role of and need for the role of physiotherapy in an ICU.

## 5. Conclusions

Physiotherapy is perceived as essential in the daily work of intensive care units, with its early approach being necessary as long as the patient’s clinical situation so requires.

Early initiation of physiotherapy treatment can help patients improve their physical and cognitive function. The shorter stay times in the intensive care unit and the early mobilization of patients are also perceived as great strengths of physiotherapy. Joint clinical sessions and fast and direct access to physiotherapists would facilitate collaboration and communication between professionals. The need to increase the number of professionals and their working hours in the ICU is described. In addition, the need to reduce the physiotherapist/patient ratio, the use of better materials and the specialization of these is described. These results apply to the study sample, and future multicenter studies should evaluate the study objectives in a larger and more heterogeneous population to confirm these findings.

## Figures and Tables

**Table 1:** The interview guides (topics and sub-topics).

*Knowledge about the role of physiotherapists in the ICU*
1. When should the physiotherapist’s work with the ICU patient begin? 2. Which is the physiotherapist’s role with these patients?
*Benefits of physiotherapy for patients admitted to the ICU and in the multidisciplinary team environment*
3. Physiotherapy-based benefits in the therapeutic approach of the ICU patient 4. Benefits that the professional considers most important to promote multidisciplinary work
*Challenges and proposals for improvement in interprofessional collaboration*
5. Challenges experienced between ICU professionals and physiotherapists 6. Proposals to improve multidisciplinary collaboration in the ICU. 7. Needs for the implementation of physiotherapy in ICU 8. Free-response remarks and comments

**Table 2:** Descriptive characteristics of the healthcare professionals included in the study.

Informant (n = 21)	Healthcare Professional	Age (Median = 40)	Sex F/M (15/6)	Working Years (Median = 10)
P01	Nursing	39	Female	10
P02	Nursing	43	Female	19
P03	Nursing	29	Female	6
P04	Nursing	33	Female	5.5
P05	Nursing	28	Female	6
P06	Physiotherapist	45	Female	2.5
P07	Internal Medicine Specialist	42	Female	11
P08	Internal Medicine Specialist	40	Female	15
P09	Internal Medicine Specialist	44	Female	19
P10	Internal Medicine Specialist	28	Female	4
P11	Internal Medicine Specialist	40	Male	14
P12	Physiotherapist	49	Male	15
P13	Internal Medicine Specialist	42	Female	12
P14	Physiotherapist	37	Female	7.3
P15	Physiotherapist	41	Female	10
P16	Nursing	36	Female	8
P17	Internal Medicine Specialist	41	Male	11
P18	Physiotherapist	34	Male	6.5
P19	Physiotherapist	40	Female	8
P20	Nursing	38	Male	10.5
P21	Physiotherapist	36	Male	5.5

**Table 3:** Issues raised by participants in the study.

Topic	Sub-Topics	Comments	Code	Category
Knowledge about the role of physiotherapists in ICU	When should the physiotherapist’s work with the ICU patient begin?	*“I think it depends on the patient, but an assessment should be made by the physiotherapist upon admission”*	P01	Nursing
		*“ In my experience, early physiotherapy is essential for improving the functional prognosis of patients”*	P16	Nursing
		*“I think passive physiotherapy benefits from the first day of admission; it usually takes several days to request it and about 24–48 h to start the treatment until evaluated by a rehabilitation specialist”*	P09	Internal Medicine Specialist
		*“As soon as the patient can cooperate minimally and is stable. Otherwise, passive physical therapy is a good way to start moving patients”*	P10	Internal Medicine Specialist
	Which is the physiotherapist’s role with these patients?	*“Their work in terms of respiratory physiotherapy is very important, they help in the weaning process, management of secretions. Also, at the motor level working on muscle strength and postural work”*	P06	Physiotherapist
		*“They can provide support with respiratory and motor physiotherapy in the ICU. Helpful for extubation. Prevention of polyneuropathies”*	P08	Internal Medicine Specialist
Benefits of physiotherapy for patients admitted to ICU and in the multidisciplinary team environment	Physiotherapy-based benefits in the therapeutic approach of the ICU patient	*“Physiotherapy shortens the days on mechanical ventilation with fewer consequences after immobilization. Better management of secretions”*	P02	Nursing
		*“ I consider the patient’s evolution to be faster and thus also an earlier improvement in their autonomy”*	P20	Nursing
		*“In general, evolution with less loss of autonomy and recovery of the latter”*	P05	Nursing
		*“ There is a better predisposition for the patient to gain autonomy in basic activities of daily living after hospitalisation”*	P19	Physiotherapist
	Benefits that the professional considers most important to promote multidisciplinary work	*“More physiotherapists, with smaller ratios. A physiotherapist permanently at the ICU the same as the rest of the staff. Multidisciplinary sessions between physiotherapists and nursing”*	P05	Nursing
		*“It is important to have a daily presence in both morning and afternoon shifts. The family’s involvement is also interesting given our extensive visiting schedule”*	P01	Nursing
Challenges and proposals for improvement in interprofessional collaboration	Challenges experienced between ICU professionals and physiotherapists	*“Communication problems due to not replacing the retainers, lifting a patient with an endotracheal tube without notifying the nurse in charge or without properly checking the venous accesses”*	P01	Nursing
		*“Collaboration hardly needs improvement, but we would need to be able to locate them with a pager for situations that require their urgent action”*	P09	Internal Medicine Specialist
		*“Following suitable training, to be able to handle the ventilators autonomously in order to work with an intubated patient”*	P12	Physiotherapist
	Proposals to improve multidisciplinary collaboration in the ICU	*“ Improve multidisciplinary communication. Clinical sessions, common lectures…”*	P14	Physiotherapist
		*“ Direct access of ICU doctors to physiotherapists for more immediate treatment”*	P13	Internal Medicine Specialist
		*“Integration of physiotherapists into the ICU team with an early intervention protocol”*	P12	Physiotherapist
Needs for the implementation of physiotherapy in ICU		*“More staff. Although it has grown a lot in recent years, they have a very large healthcare burden that does not allow them devote enough time”*	P10	Internal Medicine Specialist
		*“Support from Medical/Nursing Management. Make their importance visible. To have UCI-exclusive physiotherapists and to conduct joint sessions”*	P05	Nursing
		*“Improve the physiotherapist/patient ratio, physical therapists to be in the ICU all day in different shifts, and coordination with other services for the time of discharge from the ICU’”*	P17	Internal Medicine Specialist
		*“Clarification of the physiotherapist/patient ratio, depending on the complexity of the ICU, training of the entire team in early mobilization with complex devices and more material (foot pedals and especially crane-walker frames)”*	P12	Physiotherapist
Free-response remarks and comments		*“They are essential in daily work and in the prognosis and quality of life after admission to the ICU”*	P10	Internal Medicine Specialist
		*“They are of vital importance for the recovery of our patients. Their involvement has substantially improved the quality of care”*	P09	Internal Medicine Specialist
		*“Although the role of physiotherapy is very important in ICU patient management, there is a need for greater recognition at hospital, clinical and societal levels”*	P18	Physiotherapist
		*“They are very necessary and should be able to spend more time in the ICU”*	P08	Internal Medicine Specialist

## Data Availability

The data that support the findings of this study are available on request from the corresponding author. The data are not publicly available due to privacy or ethical restrictions.

## References

[1] Aguilar García C.R., Martínez Torres C., Aguilar García C.R., Martínez Torres C. (2017). La realidad de la Unidad de Cuidados Intensivos. Med. Crítica Col. Mex. Med. Crítica.

[2] Ervin J.N., Kahn J.M., Cohen T.R., Weingart L.R. (2018). Teamwork in the intensive care unit. Am. Psychol..

[3] Valentin A., Ferdinande P. (2011). ESICM Working Group on Quality Improvement. Recommendations on basic requirements for intensive care units: Structural and organizational aspects. Intensive Care Med..

[4] Tomonaga Y., Menges D., Yebyo H.G., Fumeaux T., Heise A., Wesch C., Schwenkglenks M., Puhan M.A. (2022). Early mobilisation and rehabilitation in Swiss intensive care units: A cross-sectional survey. Swiss Med. Wkly..

[5] Linke C.A., Chapman L.B., Berger L.J., Kelly T.L., Korpela C.A., Petty M.G. (2020). Early Mobilization in the ICU: A Collaborative, Integrated Approach. Crit. Care Explor..

[6] Schujmann D.S., Lunardi A.C., Fu C. (2018). Progressive mobility program and technology to increase the level of physical activity and its benefits in respiratory, muscular system, and functionality of ICU patients: Study protocol for a randomized controlled trial. Trials.

[7] Kho M.E., Connolly B. (2023). From Strict Bedrest to Early Mobilization: A History of Physiotherapy in the Intensive Care Unit. Crit. Care Clin..

[8] Karachi F., Gosselink R., Hanekom S. (2023). Public sector physiotherapists’ organisation and profile: Implications for intensive care service. S. Afr. J. Physiother..

[9] Jang M.H., Shin M.J., Shin Y.B. (2019). Pulmonary and Physical Rehabilitation in Critically Ill Patients. Acute Crit. Care.

[10] Inoue S., Hatakeyama J., Kondo Y., Hifumi T., Sakuramoto H., Kawasaki T., Taito S., Nakamura K., Unoki T., Kawai Y. (2019). Post-intensive care syndrome: Its pathophysiology, prevention, and future directions. Acute Med. Surg..

[11] Villamil Parra W.A., Hernández Álvarez E.D., Moscoso Loaiza L.F. (2020). Eficacia del ejercicio físico terapéutico en pacientes adultos hospitalizados en UCI: Revisión sistemática y metaanálisis. Fisioterapia.

[12] Skinner E.H., Thomas P., Reeve J.C., Patman S. (2016). Minimum standards of clinical practice for physiotherapists working in critical care settings in Australia and New Zealand: A modified Delphi technique. Physiother. Theory Pract..

[13] Takahashi T., Kato M., Obata K., Kozu R., Fujimoto T., Yamashita K., Ando M., Kawai Y., Kojima N., Komatsu H. (2020). Minimum standards of clinical practice for physical therapists working in intensive care units in Japan. Phys. Ther. Res..

[14] Resende A.S.O., Bacci S.L.L.D.S., Paula Í.R., Pereira L.A., Johnston C., Luszczynski V.C.N., Azevedo V.M.G.D.O. (2023). Performance and labor conditions of physiotherapists in Brazilian intensive care units during the COVID-19 pandemic. What did we learn?. Crit. Care Sci..

[15] Carruthers H., Derry D., Astin F. (2025). Pushing and guiding me towards home; patients’ perspectives of person-centred physiotherapy in Intensive Care. Disabil. Rehabil..

[16] Padte S., Samala Venkata V., Mehta P., Tawfeeq S., Kashyap R., Surani S. (2024). 21st century critical care medicine: An overview. World J. Crit. Care Med..

[17] Torreiro Diéguez L., Martí J.D., Souto Camba S., González Doniz L., López García A., Lista-Paz A. (2022). Fisioterapia respiratoria en Unidades de Cuidados Intensivos pediátricas y neonatales españolas. Med. Intensiva.

[18] Teherani A., Martimianakis T., Stenfors-Hayes T., Wadhwa A., Varpio L. (2015). Choosing a Qualitative Research Approach. J. Grad. Med. Educ..

[19] Jorrín Abellán I.M. (2016). Hopscotch Building: A Model for the Generation of Qualitative Research Designs. Ga. Educ. Res..

[20] Jorrín-Abellán I.M. (2019). Rayuela 2.0: Una herramienta para promocionar la labor innovadora de maestros y maestras mediante la generación de diseños de investigación rigurosos. Rev. Latinoam. Tecnol. Educ.—RELATEC.

[21] Isman E., Mahmoud Warsame A., Johansson A., Fried S., Berggren V. (2013). Midwives’ Experiences in Providing Care and Counselling to Women with Female Genital Mutilation (FGM) Related Problems. Obstet. Gynecol. Int..

[22] Guest G., Bunce A., Johnson L. (2006). How many interviews are enough? An experiment with data saturation and variability. Field Methods.

[23] Francis J.J., Johnston M., Robertson C., Glidewell L., Entwistle V., Eccles M.P., Grimshaw J.M. (2010). What is an adequate sample size? Operationalising data saturation for theory-based interview studies. Psychol. Health.

[24] Braun V., Clarke V. (2006). Using thematic analysis in psychology. Qual. Res. Psychol..

[25] Thorne S., Kirkham S.R., O’Flynn-Magee K. (2004). The Analytic Challenge in Interpretive Description. Int. J. Qual. Methods Arch..

[26] Lincoln Y., Guba E. (2004). But Is It Rigorous? Trustworthiness and Authenticity in Naturalistic Evaluation. New Dir. Program. Eval..

[27] Stiller K. (2013). Physiotherapy in intensive care: An updated systematic review. Chest.

[28] Matsuoka A., Yoshihiro S., Shida H., Aikawa G., Fujinami Y., Kawamura Y., Nakanishi N., Shimizu M., Watanabe S., Sugimoto K. (2023). Effects of Mobilization within 72 h of ICU Admission in Critically Ill Patients: An Updated Systematic Review and Meta-Analysis of Randomized Controlled Trials. J. Clin. Med..

[29] Magalhães P.A.F., Camillo C.A., Langer D., Andrade L.B., Duarte M.d.C.M.B., Gosselink R. (2018). Weaning failure and respiratory muscle function: What has been done and what can be improved?. Respir. Med..

[30] Miranda Rocha A.R., Martinez B.P., Maldaner da Silva V.Z., Forgiarini Junior L.A. (2017). Early mobilization: Why, what for and how?. Med. Intensiva.

[31] Dubb R., Nydahl P., Hermes C., Schwabbauer N., Toonstra A., Parker A.M., Kaltwasser A., Needham D.M. (2016). Barriers and Strategies for Early Mobilization of Patients in Intensive Care Units. Ann. Am. Thorac. Soc..

[32] Tadyanemhandu C., Ntsiea V., van Aswegen H. (2024). Implementation strategies to overcome barriers to early mobilisation practices in Zimbabwean and South African public sector ICUs: A Delphi study. S. Afr. J. Crit. Care.

[33] Walsh T.S., Salisbury L.G., Merriweather J.L., Boyd J.A., Griffith D.M., Huby G., Kean S., Mackenzie S.J., Krishan A., Lewis S.C. (2015). Increased Hospital-Based Physical Rehabilitation and Information Provision After Intensive Care Unit Discharge: The RECOVER Randomized Clinical Trial. JAMA Intern. Med..

[34] Barber E.A., Everard T., Holland A.E., Tipping C., Bradley S.J., Hodgson C.L. (2015). Barriers and facilitators to early mobilisation in intensive care: A qualitative study. Austral. Crit. Care.

[35] Engel H.J., Tatebe S., Alonzo P.B., Mustille R.L., Rivera M.J. (2013). Physical therapist—Established intensive care unit early mobilisation program: Quality improvement project for critical care at the University of California San Francisco Medical Center. Phys. Ther..

[36] Gruther W., Pieber K., Steiner I., Hein C., Hiesmayr J.M., Paternostro-Sluga T. (2017). Can Early Rehabilitation on the General Ward After an Intensive Care Unit Stay Reduce Hospital Length of Stay in Survivors of Critical Illness?: A Randomized Controlled Trial. Am. J. Phys. Med. Rehabil..

[37] Klarmann S., Hierundar A., Deffner T., Markewitz A., Waydhas C. (2024). Therapeutic healthcare professional staffing requirements in intensive care units. Med. Klin. Intensivmed. Notfmed..

[38] Siesage K., Schandl A., Johansson M., Nygren-Bonnier M., Karlsson E., Joelsson-Alm E. (2024). Mobilisation of post-ICU patients—A crucial teamwork between physiotherapists and nurses at surgical wards: A qualitative study. Disabil. Rehabil..

[39] Svensson-Raskh A., Schandl A., Holdar U., Fagevik Olsén M., Nygren-Bonnier M. (2020). “I Have Everything to Win and Nothing to Lose”: Patient Experiences of Mobilization Out of Bed Immediately After Abdominal Surgery. Phys. Ther..

[40] Eggmann S., Timenetsky K.T., Hodgson C. (2024). Promoting optimal physical rehabilitation in ICU. Intensive Care Med..

